# Can Integrated Agriculture-Nutrition Programmes Change Gender Norms on Land and Asset Ownership? Evidence from Burkina Faso

**DOI:** 10.1080/00220388.2015.1036036

**Published:** 2015-08-27

**Authors:** Mara van den Bold, Andrew Dillon, Deanna Olney, Marcellin Ouedraogo, Abdoulaye Pedehombga, Agnes Quisumbing

**Affiliations:** * International Food Policy Research Institute, WashingtonDC, USA; ** Department of Agricultural, Food, and Resource Economics, Michigan State University; + Helen Keller International, Ouagadougou, Burkina Faso

## Abstract

This article uses a mixed-methods approach to analyse the impact of an integrated agriculture and nutrition programme in Burkina Faso on women’s and men’s assets, and norms regarding ownership, use and control of assets. We use a cluster-randomised controlled trial to determine whether productive asset transfers and increased income-generating opportunities for women increase women’s assets over time. Qualitative work on gender norms finds that although men still own and control most assets, women have greater decision-making power and control over home gardens and their produce, and attitudes towards women owning property have become more favourable in treatment areas.

## 1. Introduction

Growing concern about the limited evidence from rigorous evaluations of nutritional impacts of nutrition-sensitive agriculture programmes has led to increased emphasis on using rigorous evaluation designs to assess and document the nutritional impact of nutrition-sensitive agriculture programmes, including assessment and understanding of the outcomes along the hypothesised programme impact pathways. Ruel and Alderman (2013), for example, identify six pathways through which agricultural interventions can impact nutrition: (1) agriculture as a source of food for own consumption; (2) agriculture as a source of income; (3) the impact of agricultural policies on prices of food and non-food crops; (4) the effect of women’s social status and empowerment on their access to and control over resources; and (5) the impact of women’s participation in agriculture on their time allocation; and (6)on their own health and nutritional status (vi).

A key factor hypothesised to influence the impact of nutrition-sensitive agricultural programmes on nutrition outcomes is their effect on increasing women’s control over assets. Assets can include (1) natural resource capital (land), (2) physical capital (livestock, agricultural assets, or household assets), (3) human capital (education, skills, health), (4) financial capital (savings, credit), (5) social capital (membership in organisations, networks), and (6) political capital (citizenship, participation). While assets can be held collectively, jointly or individually, in comparison to men, women tend to have fewer assets, have control over/ownership of different types of assets and use their assets differently (Meinzen-Dick, Behrman, Menon, & Quisumbing, 2011).

Collective models of the household (for example, Browning, Bourguignon, Chiappori, & Lechene [1994]; Browning & Chiappori [1998]) provide a theoretical framework to investigate the implications of an asset transfer to women in the context of an agricultural programme. The empirical literature generally rejects the assumption of income pooling within households (Attanasio & Lechene, 2002, 2014) or the Pareto optimality of allocation of inputs across men’s and women’s plots (Udry, 1996). However, the gender of the person who has control over assets can influence the wellbeing of household members, since intrahousehold allocation of assets affects bargaining power of individuals within households and because men and women often have different preferences for allocating resources (Alderman, Hoddinott, Haddad, & Udry, 1995; Hoddinott & Haddad, 1995; Quisumbing, 2003; Quisumbing & Maluccio, 2003). Specifically, increasing women’s control over assets has been shown to have positive impacts on food security, child nutrition, education and women’s own wellbeing (Quisumbing, 2003; Smith, Ramakrishnan, Ndiaye, Haddad, & Martorell, 2003; World Bank, 2001). Therefore, nutrition-sensitive agriculture programmes that include asset transfers can improve both women’s production possibilities and their bargaining power within the household.

Consequently, nutrition-sensitive agriculture programmes often target women under the assumption that increasing women’s control over assets in addition to improving their knowledge and skills will optimise a programme’s agricultural, health and nutritional impacts. A recent systematic review suggests that cash transfer and other similar programmes targeted to women rather than men positively impact child health and nutrition outcomes (Yoong, Rabinovich, & Diepeveen, 2012), though Doepke and Tertilt (2014) show that increasing women’s empowerment may have mixed theoretical results when production function assumptions differ.^1^ This is particularly pertinent to this study as there is limited evidence on the impact of nutrition-sensitive programmes on women’s control over assets, and existing evidence shows mixed results (Meinzen-Dick et al., 2011; van den Bold, Quisumbing, & Gillespie, 2013a).^2^

Due to gender norms, women often face more constraints than men in acquiring and using ownership rights to certain assets.^3^ Many customary systems rely heavily on family structure, inheritance and marriage practices to determine property rights. In many customary systems in Africa, women often have indirect access to land and the produce derived from it through their male relatives; yet they usually do not have full ownership rights (Gray & Kevane, 1999; Lastarria-Cornhiel, 1997).^4^ In western Burkina Faso, Kevane and Gray demonstrated that while married women from certain ethnic groups (for example, the Mossi) would farm plots independently from their husbands, having considerable control over crops grown and the income derived from them, women from other ethnic groups such as the Bwa or Lobi generally did not have these rights (Gray & Kevane, 1999).

In 2010, Helen Keller International (HKI) initiated a two-year Enhanced-Homestead Food Production (E-HFP) programme (which we refer to as ‘the programme’) in Gourma Province in eastern Burkina Faso. The ethnic groups in the programme’s treatment area, the Gourmancema and Zaoga, are generally considered to perceive women’s rights and their role in agriculture similarly to that of the Mossi, in that, women may farm plots independently and maintain control of the produce of these plots (Government of Burkina Faso, 2006). The programme’s objectives were to improve women’s production of nutrient-rich foods, their health and nutrition-related knowledge and practices, and their nutrition and health outcomes, as well as that of their children. HKI worked with communities to identify land that could be used by women for a communal farm, and provided beneficiary (participating) women with agriculture inputs and chicks to start their home production activities.

In this article we draw on van den Bold et al. [2013b], and examine how HKI’s E-HFP programme influenced women’s accumulation, ownership and control over agricultural and animal assets and norms regarding control and ownership of those assets, using a mixed methods approach. We used quantitative data from a randomised control trial to assess the short-term net effect of the asset transfer on intrahousehold asset distribution, and in turn to determine whether productive assets transferred and increased income generating opportunities for women remain with the women or are re-appropriated within the household. To disentangle the programme’s effects on women’s bargaining power, we used data from a qualitative study (using the same randomised design) that investigated the programme’s effects on gender norms. In the next section of the article, we outline the collective intrahousehold model, which illustrates how the asset transfer programme may affect intrahousehold distribution of assets, apart from the direct transfer itself. The third section presents our results, and the last section concludes.

## 2. Theoretical Framework

To motivate the analysis, we present a simple intrahousehold bargaining model that illustrates the potential effects of the asset transfer component of the treatment on women’s and men’s assets. In seminal work, cooperative intrahousehold bargaining models (for example, Basu [2006]; Browning et al., [1994]; Browning & Chiappori [1998]; Chiappori [1988, 1992]) used minimal assumptions about intrahousehold efficiency to establish how intrahousehold bargaining may affect household resource allocation. A Pareto efficient household maximises a household utility function where utility over consumption and leisure is weighted by household member Pareto weights, $\lambda_{i}$ assigned for each individual *i* within the household (Bardhan & Udry, 1999). In Burkina Faso, Udry (1996) investigates the Pareto efficiency of household labour and inputs, finding evidence in this context that households may not efficiently allocate labour and inputs across plots. This finding suggests that non-cooperative household models may better characterise intrahousehold distributions, at least in Burkina Faso.

The agricultural asset transfers from the programme could have direct effects not only on women’s production possibilities and asset accumulation over time, but also their bargaining power within the household. Men could appropriate some of the asset transfer depending on the initial bargaining power of men and women within the household. A simple non-cooperative agricultural household model has been formulated by Slootmaker (2014) to analyse how intrahousehold bargaining affects individual asset allocations. The female problem maximises an additively separable utility function of female consumption, $c_{ft}$, in period *t* and discounted utility in period *t* + 1, which is derived from the *t* + 1 female asset stock, $W_{ft+1}$. In period *t*, women choose how much to consume and invest, $x_{ft}$ with normalised price, *p*, out of their fraction of household wealth, $\pi W_{t}$. π is the female bargaining weight where $\pi \in \left[0,1\right]$. In period *t* + 1, investment in period *t* ($x_{ft}$), becomes female assets in period *t* + 1 less an intrahousehold transfer between men and women, $\Theta_{t+1}$. This maximisation problem is formalised in Equations (1)–(3), whereas the male problem is summarised in Equations (4)–(6).

(1) $$max_{c_{ft}\in \left(0,W_{ft}\right)}u(c_{ft})+\beta u\left(W_{ft+1}\right)$$

(2) $$s.t.\;W_{ft}\;=\;\pi W_{t}\geq c_{ft}+px_{ft}$$

(3) $$W_{ft+1}=f\left(x_{ft}\right)+\Theta_{t+1}$$

The male problem is:

(4) $$max_{c_{mt}\in \left(0,W_{mt}\right)}u(c_{mt})+\beta u\left(W_{mt+1}\right)$$

(5) $$s.t.\;W_{mt}\;=\;\left(1-\pi \right)W_{t}\geq c_{mt}+px_{mt}$$

(6) $$W_{mt+1}=f\left(x_{mt}\right)-\Theta_{t+1}$$

The household problem is then to maximise the Nash Product in period *t* + 1 given individually controlled assets, $\left(-W_{ft+1},W_{mt+1}\right)$ while choosing the appropriate net male to female transfer, Θ. The Nash product includes female and male indirect utilities ($V_{f},V_{m})$ from *t* + 1 wealth and indirect utility from the male or female’s best outside option ($V_{f}^{e},V_{m}^{e})$.

(7) $$max_{\Theta \in \left(-W_{ft+1},W_{mt+1}\right)}N=[V_{f}(W_{ft+1}+\Theta )-V_{f}^{e}]\left[V_{m}\left(W_{mt+1}-\Theta \right)-V_{m}^{e}\right]$$

Slootmaker (2014) characterises the individual best response when we assume logarithmic preferences $u\left(c\right)=\ln\left(c\right)$ and a linear production with productivity parameter *R*, $f\left(x\right)=Rx$ such that:

(8) $$max_{\Theta \in \left(-W_{ft+1},W_{mt+1}\right)}N=[\ln\left(\pi R\left[x_{f}+x_{m}\right]+\Theta \right)-ln\left(Rx_{f}\right)][\ln\left(\left(1-\pi \right)R\left[x_{f}+x_{m}\right]-\Theta \right)-ln\left(Rx_{m}\right)]$$

The optimal sharing rule then can be solved where $\Theta^{*}=R\left[\left(1-\pi \right)x_{f}-\pi x_{m}\right]$. Female asset holdings in any period are determined by the productivity parameter, the female bargaining weight and initial asset holdings of men.

(9) $$x_{f}=\frac{R\pi x_{m}-\Theta^{*}}{R\left(1-\pi \right)}$$

This specification motivates our mixed methods approach to understanding the effects of asset transfers on women’s asset holdings. An asset transfer changes the relative distribution of assets within the household between men and women and, hence, women’s future wealth. However, intrahousehold transfers may erode the effect of the asset transfer over time. This net effect can be measured by comparing women’s changes in asset holdings over time to measure the net effect of the transfer, the return on the asset, and the intrahousehold asset transfer to men.

This theoretical framework also motivates how qualitative research might permit us to examine one of the ‘black boxes’ of the non-cooperative model’s assumptions. One limitation with the current model is that it assumes that female bargaining share, π, is exogenous and determined by social norms. The asset transfer programme, focused on increasing women’s asset control and ownership, may affect this social norm, altering female bargaining power over time. The qualitative work was designed to investigate the plausibility of changes in this social norm due to the programme. In the next section we describe the programme and the design of both the quantitative and qualitative work.

## 3. Study Design and Methodology

### 3.1. Programme Description

The goal of HKI’s E-HFP programme in Burkina Faso was to improve maternal and child health and nutrition outcomes through a nutrition-sensitive agriculture programme targeted to women that includes a small transfer of agriculture and animal assets, training in optimal agriculture and animal-raising practices and optimal health and nutrition practices delivered through a behaviour change communication (BCC) strategy. These impacts are expected to come through three primary programme impact pathways:

1. increased availability of nutrient-rich foods through household production, primarily during the secondary agriculture season;
2. income-generation through the sale of surplus household production;
3. increased maternal knowledge and adoption of optimal health and nutrition practices.

The programme may also improve maternal and child health and nutrition outcomes through increasing women’s access to and control over resources (Ruel & Alderman, 2013). These include resources such as additional income from the sale of products from home/village production activities, improved knowledge, skills and self-confidence in agriculture, health and nutrition gained through trainings, or increases in bargaining power through the programme’s transfer of productive assets. Changes in gender norms around asset control/ownership may also influence the programme’s potential impact on agriculture, health and nutrition outcomes.

In Burkina Faso, two key components of the programme sought to directly increase women’s access to and control over agriculture-related assets. First, HKI worked with land owners in communities to identify and obtain rights to land that could be used for a Village Model Farm (VMF), run by women Village Farm Leaders (VFLs) who were beneficiaries of the programme.^5^ The VMFs served as training sites for women to learn about homestead food production and small-animal rearing, to enable them to start their own home production activities, and, in some cases, as a place to work and harvest produce.^6^ HKI supplied the VMFs with the agriculture and animal inputs (primarily chicks) at the beginning of the programme. In addition, HKI provided beneficiary women seeds, saplings, small gardening tools and chicks for their own home production activities.^7^

### 3.2. Study Design

HKI partnered with the International Food Policy Research Institute (IFPRI) to evaluate the programme’s impacts using a randomised control design coupled with two rounds of qualitative research. The quantitative longitudinal impact evaluation assessed the programme’s impact on outcomes including production, consumption, asset ownership, food security, health and nutrition-related knowledge and practices, and maternal and child health and nutrition outcomes, among others. The two rounds of qualitative research were used to understand how and why the programme did or did not have the expected impacts, as well as how the programme influenced women’s control over and ownership of productive assets, and related social norms toward this.

#### 3.2.1. Sampling: impact evaluation

**Figure 1.**
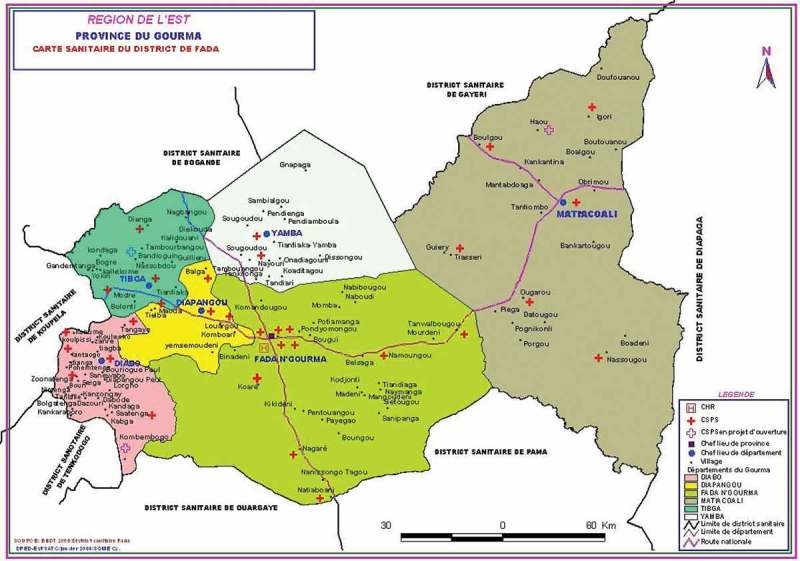
Map of study site in Gourma Province.

*Source*: Base Nationale de Données Topographiques 2000/district sanitaire Fada.

Villages were selected according to a three-step process. First, Gourma Province in eastern Burkina Faso (Figure 1) was selected because HKI had experience implementing nutrition and health programmes in this area. Within this region, four departments were selected where HKI and other non-governmental organisations did not have much prior activity, to avoid biasing results due to participation in other (possibly similar) programmes. Second, within these four departments, villages that had access to water in the dry season and were therefore capable of carrying out a gardening project were identified (N = 55). A list of households with children under 12 months of age was then compiled for each of these 55 villages. Third, after stratifying the villages by department and village size to maintain a balanced distribution of geographic locations and village sizes between treatment and control villages, villages were randomly selected into 25 control villages and 30 treatment villages. Treatment villages received gardening and small animal raising interventions and health and nutritional counselling through HKI’s BCC strategy.^8^ All households in these villages that had children 3–12 months of age at baseline (2010) were invited to participate in the programme and associated study. The baseline survey was conducted in 2010 and the endline in 2012.

#### 3.2.2. Sampling: qualitative research

Households for the qualitative research were randomly selected from each of the 30 treatment villages, and from 15 (of the 25) control villages that participated in the baseline survey. For the first round of qualitative research (2011), semi-structured interviews (SSIs) were conducted with five randomly selected households in each village included in the qualitative research. Two of the five households in each village were selected to complete a longer SSI that collected more in-depth information (Table 1). The same households participated in the first and second rounds of qualitative research to the extent possible. If a household from the first round of was not available to participate in the second round, a replacement household was randomly selected from the list of households that participated in the baseline survey.

**Table 1.** Overview of methods and participants from intervention villages and control villages

Impact evaluation			
	Intervention villages	Control villages	Total
Number of villages	30^a^	25	55
Number of households			
Baseline (2010)			
Household interview	1,023	734	1,757
Endline (2012)			
Household interview	880	590	1,470
Qualitative research			
Number of villages	29^a^	15	44
Number of households			
First round (2011)			
Basic semi-structured interviews	145	75	220
In-depth semi-structured interviews	58	30	88
Second round (2012)			
Semi-structured interviews	145	75	220

*Source*: Compiled by authors.

*Note*: ^a^One village dropped out of the programme and study before the first round of qualitative research, resulting in a total of 14 villages for the first and second rounds of qualitative research and for the endline survey for the impact evaluation. The reason for the village to drop out was a lack of social cohesion due to conflict.

**Table 2.** Gender-specific questions

Key questions	Impact evaluation	Qualitative research
Did the E-HFP program increase women’s and/or men’s ownership of assets?	√	
Did the land agreements and/or project activities influence community norms vis a vis women’s land ownership or land rights, and, if so, how?		√
Were women able to maintain control over the HFP activities and outputs as intended in the program design? What were the barriers and/or facilitators to maintaining or not maintaining this control?	√	√

*Source*: Compiled by the authors.

### 3.3. Study Methods

The units of analysis for the impact evaluation were the household as well as individuals within the household. The household head was asked to answer questions about the different household members, their health, education and dwellings. Both male and female respondents were then interviewed separately about issues including asset and animal ownership (Dillon, Moreira, Olney, Pedehombga, & Quinones, 2012). For the qualitative research, SSIs were carried out with households and key informants in treatment and control villages. In the first round, SSIs with beneficiary women covered issues related to implementation and utilisation of the programme components, perceptions of the quality of these components and barriers and enablers to inform their optimal implementation and utilisation. SSIs were also carried out with control households to establish a counterfactual. The second round of qualitative research (2012) used SSIs to understand men’s and women’s views about acquisition, use and ownership of assets and agricultural decision-making in treatment and control villages. Women were interviewed in each household, and their husbands were interviewed for selected modules.

### 3.4. Data Collection and Analysis

The baseline survey was conducted between February and April 2010 and the endline between February and May 2012 with the same households. Impacts were estimated for specific outcomes comparing results from the treatment villages to those from control villages, using a difference in differences specification controlling for baseline characteristics. The pooled specification was estimated with the following regression:

(10) $$\Delta Y_{Endline}-Y_{Baseline}=\beta Treated+\gamma X_{Baseline}+\varepsilon$$

where $\Delta Y_{Endline}-Y_{Baseline}$ is the change in programme indicator variable between the endline and baseline survey, which could be either a household-level or individual-specific indicator. Treated indicates whether the household or individual received the E-HFP programme or not (1 = treated, 0 = not treated). The specification also included baseline characteristics of the household or child depending on the programme indicator variable chosen. Balancing tests of several baseline characteristics are presented in Table A1 in the Online Appendix, and demonstrate that household characteristics such as household size and education of the head of household, as well as men’s and women’s asset values were balanced at baseline. No characteristics can be rejected at the 5 per cent level of statistical significance. The specifications still include baseline characteristics to increase the precision of estimates. The regressions were also estimated with corrections for clustering at the village level, the unit at which intervention was assigned, and for attrition using inverse probability weights (Wooldridge, 2002); see Table A2 in the Online Appendix for the attrition specification used to correct for observable attrition bias in our estimates.

The first round of qualitative data collection was carried out in May and June of 2011 and the second round in May and June of 2012. Qualitative data were manually coded by grouping similar responses together and looking for common themes among the respondents. Both the impact evaluation and the qualitative research addressed questions that aimed to understand the gender-related impacts of the programme, presented in Table 2.

## 4. Results

### 4.1. Impacts on Men’s and Women’s Land and Non-Land Productive Assets

The programme transferred agricultural assets (watering can, rake and shovels to each beneficiary) and small animals (two chickens) directly to women. In addition, 10 chickens and one rooster were given to each VMF, and in select villages six goats were given to the VMF. The VMF was also equipped with a wheelbarrow and a pickaxe. Part of the evaluation aimed to assess whether implementation of the programme resulted in changes in ownership of assets, particularly agricultural assets and small animals, by men and women, relative to the asset transfer from the programme. The impact evaluation specifically examined changes in the amount and value of household durables, agricultural assets, small animals and large livestock held by men and women between baseline and endline. To provide context for these programme impacts, descriptive statistics for household durables and agricultural assets, livestock assets and land holdings are presented in Tables 3 and 4. Difference-in-differences estimates of programme impacts are presented in Table 5. These impacts represent the compounded net effect of intrahousehold transfers changes ($\Theta^{*}$) and the endogenous change in women’s bargaining power (π) within the household. As the vector of women’s asset holdings (x) represents the portfolio of durable and agricultural assets, livestock and land, it is possible that the distribution within the portfolio may also change due to realised transfers or increased bargaining power over the composition of the asset portfolio.

**Table 3.** Household durables and agricultural assets at household level and by gender

	Baseline		Endline	
Variable	Control N = 733	Treatment N = 1,025	Control N = 597	Treatment N = 884
Household durables: number				
Men	8.4	8.3	8.8	8.8
	(7.8)	(9.5)	(9.4)	(9.6)
Women	28.86	27.1	27.8	28.9
	(18.7)	(17.1)	(21.1)	(22.0)
Household	37.3	35.4	36.6	37.7
	(22.5)	(21.0)	(26.3)	(27.6)
Household durables: value				
Men	30,207	25,672	25,892	25,689
	(41,927)	(45,788)	(33,993)	(35,030)
Women	33,137	32,067	38,370	38,277
	(34,801)	(39,475)	(39,855)	(37,684)
Household	63,344	57,739	64,262	63,966
	(63,053)	(65,191)	(63,848)	(59,950)
Agricultural assets: number				
Men	6.5^a^	7.0	8.4	8.1
	(4.3)	(5.5)	(6.8)	(6.2)
Women	2.7^a^	2.7	3.4	4.5
	(2.5)	(2.6)	(3.1)	(3.7)
Household	9.2^a^	9.7	11.8	12.6
	(5.5)	(6.3)	(8.0)	(7.7)
Agricultural assets: value				
Men	23,241^a^	23,395	28,078	24,072
	(35,524)	(47,395)	(66,709)	(36,406)
Women	1,853^a^	1,537	2,101	4,035
	(3,903)	(3,232)	(7,864)	(9,747)
Household	25,094^a^	24,932	30,179	28,107
	(35,826)	(47,583)	(67,482)	(37,477)
Total assets: number				
Men	16.3	16.6	18.8	18.4
	(11.1)	(12.8)	(14.6)	(14.1)
Women	32.3	30.3	32.2	34.3
	(20.2)	(18.3)	(23.2)	(24.0)
Household	48.5	46.9	51.0	52.7
	(26.9)	(25.0)	(32.7)	(32.9)
Total assets: value				
Men	136,995	135,171	151,839	142,843
	(168,998)	(204,070)	(223,254)	(209,021)
Women	50,196	47,468	56,395	59,797
	(47,648)	(68,765)	(52,844)	(64,305)
Household	187,191	182,639	208,234	202,640
	(190,946)	(227,503)	(250,287)	(232,384)

*Source*: Authors’ computations.

*Notes*: Numbers are mean and standard deviations (in parentheses).

All monetary values are reported in CFA francs, which are fixed to the euro in a ratio of 1 euro = 655.957 CFA francs or one CFA franc = 0.00152449 euros.

^a^ N = 732.

**Table 4.** Household livestock holdings and land cultivated by gender

	Baseline		Endline	
	Control N = 738	Treatment N = 1,025	Control N = 418	Treatment N = 730
Small animals: number				
Men	24.0	23.0	24.3	25.1
	(25.8)	(64.3)	(24.1)	(22.9)
Women	5.2	4.9	6.7	8.6
	(6.9)	(7.2)	(8.1)	(9.3)
Household	29.2	27.9	31.0	33.7
	(28.7)	(65.3)	(28.3)	(28.4)
Small animals: value				
Men	139,499	123,617	212,309	212,365
	(166,398)	(157,316)	(262,952)	(262,249)
Women	29,034	26,319	56,181	55,011
	(49,906)	(48,251)	(76,944)	(74,706)
Household	168,533	149,936	268,489	267,376
	(185,702)	(178,585)	(295,315)	(294,981)
Large Livestock: number				
Men	4.3	3.4	4.8	5.4
	(6.5)	(5.2)	(7.1)	(10.2)
Women	1.4	0.1	0.1	0.1
	(35.7)	(0.7)	(0.5)	(0.6)
Household	5.7	3.5	4.9	5.5
	(36.2)	(5.4)	(7.2)	(10.3)
Large Livestock: value				
Men	425,789	370,695	752,053	816,751
	(512,365)	(495,489)	(1,049,704)	(1,283,962)
Women	12,444	6,463	7,917	5,916
	(71,783)	(52,024)	(54,489)	(42,398)
Household	438,234	377,158	759,970	822,667
	(528,404)	(506,448)	(1,056,992)	(1,290,597)
Land cultivated (hectares)				
Men	679.0	920.0	527.0	768.0
	3.1	3.2	2.8	3.1
	(2.8)	(3.1)	(1.9)	(3.9)
Women	511.0	718.0	348.0	760.0
	1.2	1.4	1.6	0.8
	(1.8)	(5.4)	(8.2)	(1.7)

*Source*: Authors’ computations.

*Notes*: Numbers (for small animals and large livestock) are N, mean and standard deviation (in parentheses). Numbers for land are hectares, mean and standard deviations (in parentheses).

All monetary values are reported in CFA francs, which are fixed to the euro in a ratio of 1 euro = 655.957 CFA francs or 1 CFA franc = 0.00152449 euros.

**Table 5.** Difference-in-differences estimates of programme impact on the number and value of household durables, agricultural assets, and small animals, as well as land by gender of owner

	N	Treatment
Household durables: Number		
Men	1,473	−0.29
		(0.92)
Women	1,473	2.67
		(2.00)
Household durables: Value		
Men	1,473	2,352
		(4,181)
Women	1,473	65.62
		(3,398)
Agricultural assets: Number		
Men	1,473	−1.02**
		(0.41)
Women	1,473	1.08***
		(0.25)
Agricultural assets: Value		
Men	1,473	−3,388
		(3,499)
Women	1,473	2,133***
		(592)
Individual agricultural assets: Number		
Rake		
Men	1,481	−0.08
		(0.08)
Women	1,481	0.00
		(0.01)
Shovel		
Men	1,481	−0.09
		(0.07)
Women	1,481	0.02**
		(0.01)
Sickle		
Men	1,481	−0.34***
		(0.13)
Women	1,481	0.04
		(0.04)
Hoe		
Men	1,481	−0.782***
		(0.20)
Women	1,481	−0.231
		(0.22)
Pickaxe		
Men	1,481	−0.42
		(0.26)
Women	1,481	−0.12
		(0.24)
Axe		
Men	1,481	−0.17**
		(0.07)
Women	1,481	0.01
		(0.03)
Watering can		
Men	1,481	−0.08
		(0.05)
Women	1,481	0.90***
		(0.04)
Plough		
Men	1,481	−0.21**
		(0.09)
Women	1,481	0.02
		(0.02)
Small animals: number		
Men	1,146	4.35**
		(1.85)
Women	1,146	2.61***
		(0.84)
Small animals: value		
Men	1,146	29,352
		(21,437)
Women	1,146	1,979
		(6,418)
Land cultivated (hectares)		
Men	1,445	0.27
		(0.24)
Women	1,445	−0.45
		(0.41)

*Source*: Authors’ computations. * p < 0.1, ** p < 0.05, *** p < 0.01.

*Note*: Comparison is to a control group that did not receive any programme services.

All estimates controlled for clustering and attrition.

All values are coefficient (SE).

#### 4.1.1. Household and agricultural assets

At both baseline and endline, women on average owned slightly more than three times as many household durables as men in both intervention and control villages, and women’s assets had a higher average value than men’s (Table 3). However, difference-in-differences estimates indicate that the programme’s impact on either the number or value of household durables owned by men or women between baseline and endline was not statistically significant (Table 5). This is not particularly surprising, given the programme did not target household durable transfers and household durables are primarily consumption goods rather than productive assets.

Reflecting differences in portfolio composition, men owned on average about two and a half times as many agricultural assets as women at baseline in both treatment and control villages (Table 3). Both men and women in treatment and control villages increased the average number of agricultural assets owned between baseline and endline. However, the dynamics of these changes between baseline and endline differed between treatment and control villages. Our impact estimates suggest that in treatment villages the programme significantly increased the number of agricultural assets owned by women, and reduced those owned by men (Table 5).

Women and men in both control and treatment villages experienced an increase in the average value of agricultural assets between baseline and endline. At baseline, men in treatment villages held approximately 15 times the value of agricultural assets held by women in treatment villages (for control villages this ratio was 12.5). At endline, men in treatment villages still held a higher average value of agricultural assets, but the ratio between men and women had fallen from 15.2 to 6. For control villages, the male–female asset ratio changed in the opposite direction, from 12.5 at baseline to 13.4 at endline (Table 3). These changes are reflected in a significant positive impact of the programme on the value of female-owned agricultural assets, and a negative (though insignificant) impact on the value of male-owned agricultural assets (Table 5).

Table 5 also reports the increased probability due to the treatment of women holding specific agricultural assets. Women in treatment villages had a higher probability of owning shovels (1.5% more likely) and watering cans (82% more likely). This indicates that while some agricultural assets were retained by women after having been allocated by the programme (watering cans), women’s ability to control other assets within the household did not substantially change (shovels and rakes from the initial asset transfer). Men’s individual agricultural asset holdings did not significantly increase in treatment compared to control villages, except for the ownership of a plough. These individual asset data suggest some reallocation of assets within the household, but not necessarily a systematic pattern across households.

#### 4.1.2. Livestock, small ruminant and poultry holdings

The programme primarily focused on poultry in transfers to women. At both baseline and endline, men owned the majority of livestock both in terms of value and number of animals. Men in control villages held a slightly larger average number of small ruminant, poultry and large livestock at baseline than men in intervention villages, but this was reversed at endline (Table 4). Women in control villages also held a slightly higher average number of small ruminant and poultry at baseline than women in intervention villages, but this was also reversed at endline (Table 4), and only women in control villages reported owning any large livestock at baseline. Programme impacts on small ruminant and poultry ownership were statistically significant and positive for both men and women, with the differential increase for men larger than that for women (4.4 vs 2.6) (Table 5). However, programme impacts on the ownership of large livestock (data not shown) or the value of small animals were not significant. The average number of animals transferred to beneficiary women (3.73) depended not only on the programme allotment of animals, but also the biological success of the village’s first year breeding programme before transfer to beneficiaries.

#### 4.1.3. Land

Compared to women, men had a higher number of hectares of land cultivated at baseline and endline in both control and treatment villages (Table 4). On average, women in treatment villages relative to women in control villages reduced land cultivated between survey rounds, while men’s land holdings remained relatively constant. This effect is interpreted as an intensification of women’s agricultural production to higher-value horticultural products as opposed to small fields of sorghum or millet. With more intensive irrigation techniques during the dry season, the smaller land size cultivated by women due to the project should not be interpreted as a reduction in assets per se, as women have little formal legal right to land in either survey round, but as a redistribution of land cultivated across seasons and an increase in land quality due to availability of irrigation in the dry season. These quantitative results are consistent with reports from the SSIs about land use, reported in the next section. Table 5 presents the difference-in-differences impact estimates of land cultivated due to treatment status. The change in land cultivated by either men or women is not statistically different between treatment and control groups.

### 4.2. Influences on Community Norms regarding Land

#### 4.2.1. Men’s and women’s ability to inherit, own and use land

In the second round of qualitative research (2012), nearly all women and men in treatment and control villages reported that land for agricultural purposes was primarily obtained through inheritance and gifts. About half of men and women in both types of villages reported that marriage and widowhood were additional ways to obtain land. Most respondents stated that only men could inherit land, and that women were generally only able to obtain land through marriage or gifts.

More than half of men and women in both types of villages reported that women could not inherit land upon their husband’s death, although this was slightly higher for respondents in control villages. The overwhelming reason was reported to be due to traditional inheritance and usage rules. Mature children would inherit land from their father, or, if the land traditionally belonged to the husband’s family, they would reclaim it. Wives not native to the village in question were ‘strangers’ and could therefore not inherit. A woman reportedly only inherited land from her deceased husband if she had young children with him, if she wanted to stay in the home/family, if she was too old to marry another man, or if her husband’s family allowed her to.^9^ One woman from a treatment village reported that ‘Social considerations prevent women from inheriting land from her husband if she does not have children or if she has only girls.’ Vice versa, around half of men and women in both types of villages reported that men would usually inherit the land if their wife died. More men and women in treatment than in control villages reported that it depended upon the agreement that was in place with the previous owner.

Respondents in both types of villages reported that there were significant obstacles for women to own land, but not for men. More than half of men and women in treatment villages compared to about half of women and men in control villages reported that women faced obstacles, and that these primarily related to traditional/social norms. A woman in a treatment village stated: ‘Tradition reduces the chances for women to own land.’ Another obstacle to women owning land was that women were regarded as ‘strangers’ or ‘nomads’. Inheritance rules, mindset/non-involvement of women in land issues, and lack of fertile lands were reported as additional obstacles.

#### 4.2.2. Changes in women’s’ ability to own and use land as well as changes in gender norms

Despite the obstacles to women’s ability to own land described by respondents in our study area, a greater proportion of male and female respondents in treatment compared to control villages noted that there had been changes in women’s ability to own and use land and in opinions regarding these issues over the two-year E-HFP programme period (Table 6).^10^

More than half of men and women in treatment villages compared to less than one-quarter of men and women in control villages explained that their opinions about who can own/use land for production of fruits and vegetables had changed during the two years in which HKI was operating its programme (Table 6). The respondents who had changed their opinions attributed this to changes in gender roles, the HKI intervention, changes in consumption through enhanced understanding of the importance of consuming fruits and vegetables for nutrition and dietary diversity, and the ability to now grow fruits and vegetables in the dry season. The most frequently reported reason for changing opinions was a change in gender roles in favour of greater resources controlled by women, with respondents emphasising women’s ability to use/manage land or women’s ability – as a result of the programme – to manage and own a garden. The programme was specifically noted as a primary reason for the changes in opinion among respondents from intervention villages. One (male) respondent in a control village stated that: ‘Thanks to HKI, I realised that a woman can garden. And the case of the VMF convinced me of the benefit.’ Another male respondent reported that ‘women proved that they had the capability to manage the land well’. Those who stated that their opinions had not changed reported that one key reason was tradition/custom, as well as issues related to traditional gender roles, mainly with regard to rules around ownership.

**Table 6.** Reported changes in opinion on ownership and use of land among men and women in intervention villages over the period 2010–2012, based on semi-structured interviews

	Women		Men	
	Treatment	Control	Treatment	Control
	n = 145	n = 75	n = 114	n = 60
Change in own opinion about who can own and/or use land for the production of fruits and vegetables changed	95 (67)	11 (16)	68 (60)	14 (23)
	n = 112	n = 65	n = 97	n = 52
Perceived changes in other people’s opinions about who can own and/or use land for the production of fruits and vegetables	55 (49)	8 (12)	45 (46)	5 (10)
	n = 136	n = 73	n = 116	n = 60
Perceived changes related to women’s ability to own land in the village	33 (24)	1 (1)	31 (27)	2 (3)
	n = 138	n = 74	n = 108	n = 61
Perceived changes related to women’s ability to use land for growing food in the village	61 (44)	3 (4)	48 (44)	1 (2)

*Source*: Authors’ computations.

*Note*: Numbers are N, with percentage reported in parentheses. All of the differences were significant at *p* < 0.001.

In addition to changes in their own opinions, a greater proportion of men and women in treatment villages saw changes in other people’s opinions compared to those in control villages over the life of the programme (Table 6). Those who had seen changes in other people’s opinions observed that women increasingly had more access to land for gardening and explained that non-beneficiaries in treatment villages had been copying programme participants. For example, a participant in one of the treatment villages stated that ‘non-beneficiary men and women copied the idea and set up homestead gardens’. Another stated that ‘land owners and household heads now give land [to women] for gardens’.

In accordance with changes noted in opinions, similar trends were seen in reported actual changes in women’s ability to own land among respondents in treatment as compared to control villages. While approximately one-quarter of both female and male respondents from treatment villages stated that women’s ability to own land had changed between 2010 and 2012, there was minimal change in control villages (Table 6). Those who had seen changes in women’s ability to own land (treatment) discussed that changes mainly related to husbands and/or HKI granting land to women, and that women now had community gardens. As one man (treatment village) explained, ‘Thanks to HKI, women gain access to land when they ask for it’ and ‘Thanks to HKI, the women are owners of the VMF.’ Women from treatment villages reported that: ‘The women possess more and more land granted by their husbands’ and that ‘women gain more and more land due to the presence of the HKI project’. In contrast, no differences were noted for men’s ability to own and use land for agricultural purposes during the programme period in either treatment or control villages.

Similarly to changes reported in women’s ability to own land, close to half of men and women in treatment villages explained that there had been changes over the past two years in women’s ability to use land for growing food (Table 6). The main reported changes related to support with agricultural inputs and equipment/tools (in treatment villages only), and increased access to land for women due to transfers from men, and due to advocacy. Although study findings suggest that women’s ability to use land for agricultural purposes changed in treatment villages, women still face obstacles to using land for farming. Some of these are similar to the challenges faced by men – the lack of cultivable lands, unfavourable rainfall or lack of agricultural inputs – but obstacles also relate to traditional women’s roles. When asked about ways to improve women’s ability to use land, respondents proposed support with inputs (seeds, fertiliser) and agricultural materials/tools; these suggestions were more commonly made in treatment villages. Furthermore, sensitisation of all stakeholders was mentioned by respondents in both treatment and control villages, with government and local authorities and NGOs expected to play key roles.

Close to half of men and women in both types of villages said they expected to see changes in the way in which women will be able to own/use land in the future. Those who expected changes mostly reported that women are expected to gradually gain more access to land with support from NGOs and the government. Around a third of all respondents believed that in the future women will be able to acquire land through purchase/lease. Another expected change, reported more by those in treatment than control villages, was that women will gain more access to land through sensitisation of stakeholders.

Those who did not expect changes in women’s ability to own/use land, primarily in control villages, cited traditional practices as the primary barrier, as well as women’s lack of land rights and women’s dependence on men.^11^ Interestingly, both men and women (though more so in control villages) expected that customary rules around gifts and inheritance of land would cease to be as important in the future.

### 4.3. Control over E-HFP Activities and Outputs

The two rounds of qualitative research also aimed to assess whether women participating in the programme had been able to maintain control over programme activities and outputs. The results reported below are based on SSIs with women in treatment villages.

#### 4.3.1. Garden and produce

In 2011, 84 per cent of women in treatment villages had a home garden and for 90 per cent of these women this had occurred since they joined the programme in 2010. By contrast, only 4 per cent of women in control villages had a home garden in 2011. In 2012, 81 per cent of women in treatment villages reported that they had land near their home that was used to grow fruits and vegetables, compared to 5 per cent of women in control villages.

**Figure 2.**
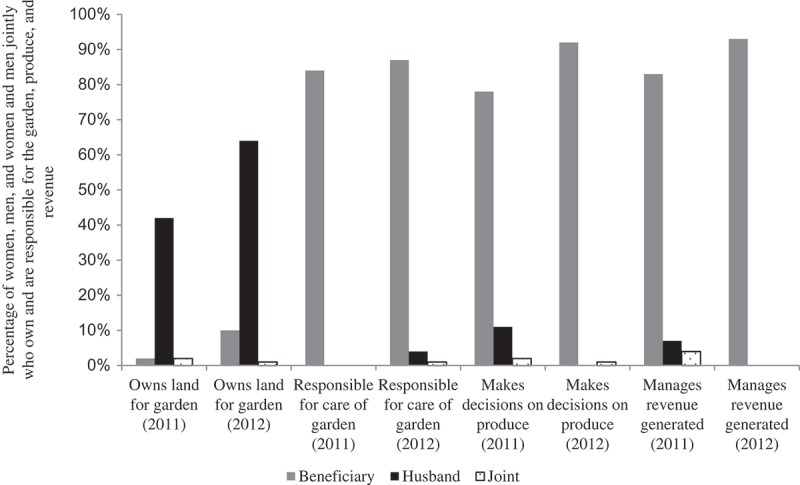
Ownership and responsibility for the home gardens, produce and revenue generated from produce as reported by beneficiary women in 2011 and 2012.

*Source*: Authors’ computations.

Land on which the garden was established was mainly owned by men, and this had actually increased from 44 per cent in 2011 to 64 per cent in 2012, possibly due to an increase in overall household ownership of land. Although women were unlikely to report that they owned the land on which gardens were established, there was a small increase in the proportion of women who reportedly owned this land from 2011 to 2012, from 2 per cent in 2011 to 10 per cent in 2012. Joint ownership remained the same at 2 per cent in 2011 and 1 per cent in 2012 (Figure 2).

Beneficiary women remained primarily responsible for the care of the garden between 2011 and 2012. In addition, they reportedly were responsible for making most decisions regarding produce from the garden, and they were also the main managers of the revenue generated from the sale of this produce. Between 2011 and 2012, women’s decision-making on produce increased from 75 per cent to 92 per cent, while male decision-making decreased from 9 per cent to 0 per cent. Similarly, the percentage of women who managed revenue from the sale of produce increased from 83 per cent in 2011 to 93 per cent in 2012, while male management decreased from 7 per cent to 0 per cent (Figure 2).

The vast majority of respondents in treatment villages expected that the land dedicated to growing fruits and vegetables would continue to be used for this purpose beyond the life of the project. About 28 per cent of women in treatment villages said that they believed that the land would always be dedicated to growing fruits and vegetables in the future because it provided the household with this particular produce, about 24 per cent of women explained that they saw it as a very beneficial activity, and about 21 per cent of women said that they would continue to grow fruit and vegetables for financial reasons.

#### 4.3.2. Chickens

**Figure 3.**
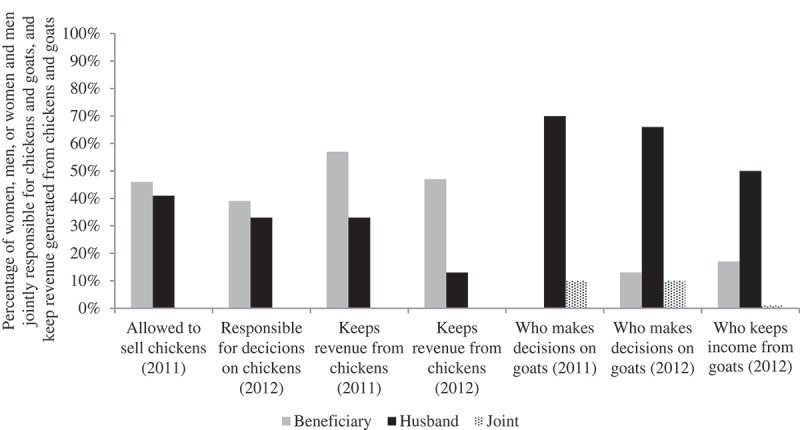
Decision-making on chickens and goats and revenue generated from chickens and goats as reported by beneficiary women in 2011 and 2012.

*Source*: Authors’ computations.

More women (46%) than men (38%) in treatment villages were allowed to sell chickens in 2011. In 2012, when a slightly different question was asked, more women (41%) than men (35%) in treatment villages were responsible for decision-making on chickens, in contrast to 29 per cent of women and 58 per cent of men in control villages. Although the proportion of women who kept the income from the sale of chickens decreased between 2011 and 2012 from 54 per cent to 48 per cent, there was also a decrease for the proportion of men who kept the revenue from the sale of chickens, from 35 per cent to 14 per cent. Despite the overall decrease in absolute percentages, the changes reflect an overall increase in the likelihood of women keeping the income from the sale of chickens as compared to men (Figure 3).

Nearly all the men and women in treatment and control villages who had land close to home used to raise chickens reported that they planned to continue to raise chickens on this land. Consumption benefits were a key reason (consumption of the meat and the eggs, and the benefits of this for children) for around 95 per cent of women in treatment villages, and about 83 per cent of women reported reasons related to improved revenue from the sale of the chickens.

#### 4.3.3. Goats

Men were in charge of decision-making and retained the income from the sale of goats. In 2011, women in treatment villages reported that 68 per cent of decisions on goats were taken by men, while joint decision-making was at 10 per cent and women did not make any decisions. In 2012, men took most of the decisions at 66 per cent; however, there was a notable increase for women from 0 per cent in 2011 to 13 per cent in 2012. There was no change in joint decision-making, which remained at 10 per cent. In 2012, 50 per cent of women in treatment villages reported that the revenue from the sale of goats was kept mainly by men, although 17 per cent of women kept income from the sale of goats (Figure 3).

Almost all who reported having land close to their home for raising goats stated that they expected to continue using the land for this purpose. Around 90 per cent of women in treatment villages expected this because goats were considered a source of revenue, while around 25 per cent of women in these villages expected this because goats were intended for consumption.

## 5. Summary and Conclusions

This article examined whether a homestead food production programme had any impact on asset ownership and control by men and women, and whether social norms regarding asset ownership and control changed as a result of the programme.

Results indicate that men continued to have the majority of control over and ownership of land and assets in both treatment and control villages in this area in eastern Burkina Faso. However, the evidence also points to shifting patterns with regard to women’s control and ownership of assets (including land) – both in terms of quantifiable changes in number and value of assets, as well as changes in communities’ perceptions and opinions that respondents in treatment villages attributed to the E-HFP programme. Some respondents also noted that non-participants had started to ‘copy the gardens’, indicating the potential for spillover effects. These changes in opinion regarding women’s land ownership suggest, qualitatively, that the programme has had some impact on perceptions of women’s land rights in these villages. This is potentially an important change in social norms, as evidence suggests that more secure land rights contributes to women controlling the income from their cultivated land and an increased willingness to invest in land (Fenske, 2011), such as increased tree planting (Goldstein & Udry, 2008; Quisumbing et al. 2001Goldstein & Udry, 2008), and the adoption of soil conservation techniques (Deininger, Ali, & Yamano, 2008).

From our theoretical model, women’s asset holdings could have been affected by either net asset transfers due to the programme or changes in bargaining power within the household. The programme effect on increased asset control for women as a net effect of the programme’s asset transfer and the increase in bargaining power to women were investigated using both quantitative and qualitative data. We found positive impacts of the programme on both the number and value of women’s agricultural assets and the number of small animals owned, although men’s holdings of small animals increased more and the number of their agricultural assets decreased (almost one-to-one with the increase in women’s agricultural assets). This indicates that there was a shift in the pattern of ownership which narrowed the proportional gap in ownership of agricultural assets between men and women in treatment villages. This did not happen in control villages. The significance of this relatively small increase in ownership of agricultural assets and the shift in ownership pattern is unclear, but is consistent with the programme’s intention to transfer small agricultural assets to women.

Accompanying the positive changes noted above, women in treatment villages also reported being able to control their gardens, the use of the products, and being able to manage the income generated from their gardens, although men maintained control of higher-value animals. Again, the implications of these relatively small changes and differences in women’s decision-making power are unclear, but they may have positive impacts on critical outcomes such as food security, child nutrition and education, and women’s own wellbeing (Quisumbing, 2003; Smith et al., 2003; World Bank, 2001). These findings are consistent with other studies of value chain projects in Malawi and Uganda, where commodities generating lower average revenues are more likely to be controlled by women, whereas men control commodities that are high revenue generators, often sold in formal markets (Njuki, Kaaria, Chamunorwa, & Chiuri, 2011).

Because the E-HFP was a two-year pilot programme, it remains to be seen whether these changes in asset ownership and control will be sustained. Nevertheless, these results point to the potential of nutrition-sensitive agricultural programmes to improve women’s control over and ownership of assets, and to change gender norms around land and asset use, control and ownership. These positive changes, in turn, could be related to positive changes in maternal and child health and nutrition outcomes in the short or long term (Ruel & Alderman, 2013).

## Disclosure statement

No potential conflict of interest was reported by the authors.

## Supplementary Material

Online appendix.pdf

## Notes

1. Although there is a large literature on different types of unearned transfers, most studies do not compare outcomes of a transfer made to women versus men (Yoong et al., 2012, p. 16). In total, only 15 studies examining five types of transfers (conditional cash transfers, unconditional cash transfers (including child benefits and old-age pensions) and cash and in-kind grants to microenterprises and micro-credit) were included in this systematic review.

2. While some studies on the impacts of agricultural interventions on women’s empowerment have demonstrated increases in women’s (control over) income or participation in household decision-making, others have not found such impacts, or have found mixed results (van den Bold et al., 2013a). In some cases, men saw higher increases in income compared to women, maintained control over income, household resources, and higher value assets, or took over control of certain assets as they became more profitable (Carney 1988; von Braun and Webb 1989).

3. For example, Lastarria-Cornhiel (1997) discusses implications of various customary land tenure systems across Africa for men’s and women’s land rights; in some societies, women may own land, but are prohibited from ploughing (Mogues et al., 2009). Doss and Deere (2006) review studies that examine gender asset gaps and reasons why these gaps persist.

4. While access and withdrawal are usually considered to be ‘use rights’, exclusion, management and alienation are considered to be more empowering ‘control rights’. ‘Ownership’ encompasses both of these ‘bundles of rights’ (Meinzen-Dick et al., 2011).

5. Traditionally, the VMF was run by a household that had a large piece of land and experience with farming, but this model tended to favour male farmers who ran the VMF as a business rather than as part of a community development programme (Hillenbrand, 2010).

6. Each VMF was approximately 1500 m^2^; a small number of villages could not accommodate this space and had VMFs of 1,000–1,200 m^2^.

7. Each beneficiary woman received two chickens from the programme, and each VMF received 10 chickens and one rooster. In five villages, each VMF also received six goats. The average number of animals transferred to beneficiary women depended not only on the programme allotment of animals, but also the biological success of the village’s first year breeding programme before transfer to beneficiaries.

8. Within the treatment group, the BCC strategy differed among two equal subgroups of the treatment (15 villages each). The two groups of intervention villages differed only by who delivered the health and nutrition counselling to the beneficiary women. In one group of 15 treatment villages, the BCC strategy was carried out by Older Women Leaders, and in the other group of 15 treatment villages it was carried out by village Health Committee members. The agricultural intervention was similar across all treatment villages and as such we pool them for the purposes of this article.

9. A woman was not able to inherit the land if she wanted to remarry outside of the husband’s family, although this was reported more among women than among men. Furthermore, women were unable to inherit land from their husbands if they had no children with him (van den Bold et al. 2013b).

10. T-tests (not reported here) indicate that for all outcomes reported in Table 6, differences between intervention and control villages are significant at p < 0.001.

11. A significant proportion of men and women in both types of villages also expected to see changes in the way in which men were able to own/use land in the future. Changes were expected to occur mainly in the ability to purchase or lease land and government distribution of lands, as well as an expectation that land scarcity will make access to cultivable lands increasingly difficult. Both men and women in participating villages expected that some form of land development and redistribution/subdivision of land would occur. Those who did not expect any future changes in men’s ability to own and/or use land cited traditional practices as the main reason (van den Bold et al. 2013b).
