# Supplementary material for: Can Integrated Agriculture-Nutrition Programmes Change Gender Norms on Land and Asset Ownership? Evidence from Burkina Faso
Source: J Dev Stud. 2015 Aug 27;51(9):1155–74. doi: 10.1080/00220388.2015.1036036 (PMC6183935; doi:10.1080/00220388.2015.1036036)
Supplement: Online appendix.pdf [file FJDS_A_1036036_SM4922.pdf]

# Can Integrated Agriculture-Nutrition Programs Change Gender Norms on Land and Asset Ownership? Evidence from Burkina Faso

MARA VAN DEN BOLD*, ANDREW DILLON**, DEANNA OLNEY*, MARCELLIN OUEDRAOGO†, ABDOULAYE PEDEHOMBGA† & AGNES QUISUMBING*

*International Food Policy Research Institute, Washington DC, USA, **Department of Agricultural, Food, and Resource Economics, Michigan State University, †Helen Keller International, Ouagadougou, Burkina Faso

## Online Appendix

**Table A1.** Household characteristics at baseline

| Variable | Pooled treatment | Control | p-value |
|---|---|---|---|
| Number of household observations | 1,026 | 741 | |
| HH size (residents present at least 6 months) | 7.23 | 7.64 | |
| | (3.59) | (3.79) | 0.28 |
| Female Headed HH | 3% | 2% | 0.67 |
| Men's asset value (FCFA) | 71,361 | 72,607 | |
| | (59,828) | (58,228) | 0.94 |
| Women's asset value (FCFA) | 44,605 | 46,607 | |
| | (66,361) | (39,594) | 0.66 |
| Men's livestock value (FCFA) | 494,576 | 565,513 | |
| | (577,336) | (612,447) | 0.22 |
| Women's livestock value (FCFA) | 32,799 | 41,525 | |
| | (77,624) | (101,784) | 0.09 |
| Dirt floor in primary house (%) | 45% | 34% | 0.06 |
| Roofing material is straw mat in primary house (%) | 59% | 58% | 0.92 |
| Education of HH head (% completed) | | | |
| Any primary or secondary education | 11% | 12% | 0.82 |
| Education of woman (% completed) | | | |
| Any primary or secondary education | 7% | 8% | 0.77 |

**Table A2.** Probability of attrition probit

| | Probit |
|---|---|
| treatment==OWL | 0.14 |
| | [0.09] |
| treatment==HC | 0.21** |
| | [0.09] |
| Number of men in HH | 0.08 |
| | [0.05] |
| Number of women in HH | –0.05 |
| | [0.04] |
| Number of boys in HH | 0.10*** |
| | [0.03] |
| Number of girls in HH | 0.07*** |
| | [0.03] |
| HH assets count | –0.00 |
| | [0.00] |
| HH livestock count | 0.00 |
| | [0.00] |
| district==Diabo | –0.19 |
| | [0.13] |
| district==Diapangou | –0.41*** |
| | [0.14] |
| district==Tibga | –0.09 |
| | [0.14] |
| Constant | 0.84*** |
| | [0.17] |
| R-squared Observations | 1767 |

*Notes*: Standard errors are reported in brackets.
* $p < 0.1$, ** $p < 0.05$, *** $p < 0.01$.
Within the treatment group, the BCC strategy differed among two equal subgroups of the treatment (15 villages each). The two groups of treatment villages differed only by *who* delivered the health and nutrition counselling to beneficiary women. In one group of 15 intervention villages, the BCC strategy was carried out by Older Women Leaders (OWLs) and in the other group of 15 intervention villages it was carried out by village Health Committees (HCs).
